# Clinical significance of small molecule metabolites in the blood of patients with different types of liver injury

**DOI:** 10.1038/s41598-021-91164-9

**Published:** 2021-06-02

**Authors:** Hui Li, Yan Wang, Shizhao Ma, Chaoqun Zhang, Hua Liu, Dianxing Sun

**Affiliations:** 1 The Liver Disease Center of PLA, The 980th Hospital of PLA Joint Logistics Support Force, Shijiazhuang, 050082 China; 2 Hebei Insitute for Drug and Medical Device Control, Shijiazhuang, 050299 China; 3 Chengde Medical University, Chengde, 067000 China; 4 Hebei Medical University, Shijiazhuang, 050000 China

**Keywords:** Cancer metabolism, Molecular biology

## Abstract

To understand the characteristic of changes of serum metabolites between healthy people and patients with hepatitis B virus (HBV) infection at different stages of disease, and to provide reference metabolomics information for clinical diagnosis of liver disease patients. 255 patients with different stages of HBV infection were selected. 3 mL blood was collected from each patient in the morning to detect differences in serum lysophosphatidylcholine, acetyl-l-carnitine, oleic acid amide, and glycocholic acid concentrations by UFLC-IT-TOF/MS. The diagnostic values of four metabolic substances were evaluated by receiver operating characteristic (ROC) curve. The results showed that the optimal cut-off value of oleic acid amide concentration of the liver cirrhosis and HCC groups was 23.6 mg/L, with a diagnostic sensitivity of 88.9% and specificity of 70.6%. The diagnostic efficacies of the three substances were similar in the hepatitis and HCC groups, with an optimal cut-off value of 2.04 mg/L, and a diagnostic sensitivity and specificity of 100% and 47.2%, respectively. The optimal cut-off value of lecithin of the HBV-carrier and HCC groups was 132.85 mg/L, with a diagnostic sensitivity and specificity of 88.9% and 66.7%, respectively. The optimal cut-off value of oleic acid amide of the healthy and HCC groups was 129.03 mg/L, with a diagnostic sensitivity and specificity of 88.4% and 83.3%, respectively. Lysophosphatidylcholine, acetyl-l-carnitine, and oleic acid amide were potential metabolic markers of HCC. Among them, lysophosphatidylcholine was low in the blood of HCC patients, and its diagnostic efficacy was better than that of acetyl-l-carnitine and oleic acid amide, providing reference metabolomics information in clinical diagnosis and future research.

## Introduction

The liver plays a vital role in overall metabolism in the body^1^. Once a disease occurs, the metabolic function will have varying degrees of liver injury, causing multiple systemic metabolic disorders in the body^2^. According to reports of the World Health Organization (WHO), the number of people infected with the hepatitis B virus (HBV) in the world population was two billion^3^. Among them, 240 million people had chronic HBV infections^4^; approximately 2–10% of patients with chronic hepatitis B (CHB) progress to cirrhosis in each year^5^; and 3–5% patients with compensated HBV cirrhosis (CHC) progress to decompensated HBV cirrhosis (DHC). The five-year survival rate of DHC patients was only 14–35%^5^, which was mainly due to the insufficiency of the early diagnosis techniques^6^.

The diagnosis of hepatocellular carcinoma (HCC) has relied on histological examination or clinical and imaging examinations^7^. However, the early diagnostic method of HCC is very limited, with poor sensitivity and specificity^8^. HCC cases with a clear diagnosis usually progress to intermediate to advanced stages with poor prognosis. Serum markers such as alpha-fetoprotein (AFP) and gene markers such as the human cervical cancer oncogene (*HCCR*) have been most widely used as HCC serological markers^9^ and are generally based on biological detection methods, such as enzyme-linked immunoreactivity, with weak specificity and high sensitivity. The most commonly used AFP marker has a sensitivity of only 61% for the diagnosis of HCC^10^. In addition, the levels of traditional serum markers are not completely and positively correlated with tumor tissue development or cancer tissue size^11^. Although a variety of imaging examinations have been applied in clinical practice^12^, the HCC diagnostic results are not very satisfactory. Furthermore, the expensive equipment and the relatively complicated diagnostic models cannot be used for screening HCC in poverty areas, which usually have high incidences of HCC. Therefore, a reproducible, high-accuracy, and low-cost test is an urgent need for the early diagnosis of HCC.

Metabolomics^13^ uses small-molecule metabolites as research objects, using high-precision analytical methods and bioinformatics tools to target all small-molecule metabolites (< 1000 relative molecular mass) in all tissues and cells for quantitative analysis^14^, which directly reflects the biochemical state of the tissues and more sensitively describes the changes in the physiological and pathological characteristics of the body^15^, expanding the screening range of markers of HCC characteristics. Compared with the traditional screening tools, the specimens for metabolomics are easily obtained, and the operation of metabolomics is simple. The research objects of metabolomics are the downstream products of genes and messengers, with relatively few types. Metabolomics simultaneously processes a large number of specimens of the patients, and the metabolites have versatility^16^, i.e., they can be indicators of HCC development and progression in the early stages and solve the problem of the existing diagnostic techniques for early HCC are not good enough.

Application of metabolomics to study the small-molecule metabolites in the blood of patients with liver injury and their underlying pathogenic mechanism is a popular research topic. Many researchers have applied nuclear magnetic resonance and tandem mass spectrometry combined with metabolomics theory to study small-molecule metabolites (e.g., amino acids, choline, and phospholipids) in the blood samples of different liver diseases, such as hepatitis B, fatty liver, cirrhosis, liver failure, and HCC^17–21^. Using random forest algorithm, difference analysis, and receiver operating characteristic (ROC) curve analysis, the application of small-molecule metabolites in the diagnosis of liver diseases was studied, and the pathogeneses of different liver diseases were further explored. In the early stage of this study, ultrafast liquid chromatography coupled with ion trap time-of-flight mass spectrometry (UFLC-IT-TOF/MS) was used to establish a screening method for broad-spectrum small-molecule metabolites in the serum of patients with HBV positive patients, including HBV-IaC, CHC, DHC, HCC, et al^22^. Twelve kinds of characteristic substances, including glycocholic acid, glycerophosphorylcholine, acetylphenylalanine, oleamide, acetyl-l-carnitine, and lysophosphatidylcholine (lysoPC) were found. Multifaceted analysis showed that four substances, including lysoPC, acetyl-l-carnitine, oleamide, and glycocholic acid, had significant characteristics. However, the clinical significance of these small-molecule metabolites and their diagnostic sensitivities as potential diagnostic markers have not been reported. This study used UFLC-IT-TOF/MS to detect the changes in the metabolite concentrations in different stages of liver diseases after HBV infection, providing new methods and markers for the early diagnosis.

## Materials and methods

### Screening of different groups of livery injury at different stages of HBV infection

This study randomly included 31 HBV carriers, 37 cases of chronic HBV, 83 cases of HBV-related cirrhosis (51 cases of CHC and 32 cases of DHC), and 46 cases of HCC treated in the 980^th^ Hospital of Chinese People’s Liberation Army Joint Logistic Support Force (Hebei Province, China) from June 2015 to October 2016. The diagnoses of the patients were in accordance with the Guidelines for the Prevention and Treatment of Chronic HBV in China published in 2015 and the Diagnostic Standard for HBV Infection issued and implemented by the Ministry of Health of the People’s Republic of China. The exclusion criteria of this study for patients were: (1) autoimmune disease; (2) liver disease(s) due to other causes, such as fatty liver, alcoholic liver disease(s), or drug-induced liver disease(s); (3) underlying accompanied diseases, such as diabetes, hypertension, lung disease, heart disease, cerebrovascular disease, and kidney disease; (4) concurrence of hepatitis C virus infection; (5) with fever above 37.5 °C; (6) other disease(s) and medical history that the researchers believed that might affect the experimental results; and (7) any other condition that the researchers believed was not suitable for the inclusion.

### Healthy control screening

A total of 58 healthy volunteers who underwent physical examinations at the Physical Examination Center of the 980th Hospital of the Chinese People’s Liberation Army Joint Logistic Support Force from June 2015 to October 2016 were included in this study as the healthy control group. The screening criteria were: (1) healthy volunteers with their liver functions and AFP within the normal reference intervals; (2) candidates without respiratory diseases (e.g., tuberculosis), endocrine diseases (e.g., diabetes), cardiovascular diseases (e.g., hypertension), kidney diseases (e.g., kidney failure), and circulatory system diseases (e.g., leukemia); and (3) candidates with no positive clinical symptoms, no viral hepatitis infection, no smoking and alcohol consumption history, and age within 20–65 years old.

### Liver function markers and demographic characteristics

All participants were asked to register their demographic information, such as name, gender, age, time of hospital visit, and clinical/hospital number, which was followed by testing and recording their biochemical and hemagglutination indices, mainly including glutamic-pyruvic transaminase (ALT), glutamic oxalacetic transaminase (AST), total bilirubin (TBil), albumin (ALB), and prothrombin time (PT) (Table 1). The ALT, AST, ALB, and TBil were detected by a P800 module automatic biochemical analyzer (Roche).

**Table 1:** Demographic information and liver function of study population.

	Control group (n = 78)	HBV-IaC (n = 28)	CHB (n = 48)	CHC (n = 30)	DHC (n = 55)	HCC (n = 116)	
Gender (male/female)	58/20	19/9	34/14	24/6	47/8	60/26	
Age (years)	37(17)^#^	35(33)^#^	41.69 ± 10.39*	43.5(12)^#^	47.36 ± 14.21*	56.5(13)^#^	
HBsAg (negative/positive)	Negative	Positive	Positive	Positive	Positive	Positive	
ALT(U/L) (3–50)	12.1 ± 2.73*	22.75(10.75)^#^	126.4(147.3)^#^	24.2(18.05)^#^	39.4(40)^#^	54.22 ± 23.29*	
AST(U/L) (3–50)	16(11.75)^#^	18.9(11.57)^#^	45(64.5)^#^	50.86 ± 23.67*	19(17)^#^	34.05(31.8)^#^	
TB (umol/L) (1–22)	8(5.25)^#^	8(9.5)^#^	21(19)^#^	25.05 ± 6.93*	24(13)^#^	24.5(18.75)^#^	
ALB (g/L) (35–55)	46(3)^#^	45.5(4.5)^#^	41.77 ± 4.03*	39 ± 3.69*	29(7)^#^	34(5)^#^	
PT(s) (10.5–14.0)	–	11.6(1.08)^2^	11.73 ± 0.73^1^	12.56 ± 0.81^1^	15.6(3.1)^2^	13.5(2.95)^2^	

*Data as normal, described as: mean +/−standard deviation,* P* > 0.1; #data for the non-normal, described as: the median (interquartile range), *P* < 0.1;– : not to do.

### Experimental materials

#### Standard preparation

The ion mode detected by each substance was determined according to the corresponding value of the standard substance mass spectrometer. Table 2 shows the name and ion mode of each compound. The concentration of the standard stock solution was 5.0 mg/mL. The solvent was the mobile phase 0.1% formic acid aqueous solution (A)-acetonitrile (B), followed by diluting the concentration of each compound as 25 mg/L mixed standard working solutions, which were used for comparisons between the different preprocessing steps and methodological investigation. The internal standard solution was 2-chlorophenylalanine at a concentration of 0.1 mg/mL.

**Table 2:** List of reference standards and content and difference analysis of compounds in different group.

NO	Metabolite	CAS number	Ion mode	Relative molecular weight	Healthy group	HCC group	*P* value
					Content (mg/kg)		
1	Glycocholic acid	1192657-83-2	ESI+	466.3212	0	2.148	*P* < 0.01
2	Glycerophosphorylcholine	28319-77-9	ESI−	258.1315	0.263	0	*P* < 0.01
3	Acetylphenylalanine	2018-61-3	ESI−	207.2526	0	0.573	*P* < 0.01
4	Oleamide	301-02-0	ESI+	282.2813	2.591	9.588	*P* < 0.01
5	Myristamide	–	ESI−	228.2375	0.221	0.266	*P* < 0.01
6	Phenylacetylglutamine	28047-15-6	ESI−	265.1358	3.409	2.467	*P* > 0.05
7	7-Methylguanine	–	ESI−	166.0756	1.245	1.383	*P* > 0.05
8	7-[(1S, 2S)-2-(Heptylamine) cyclohexyl] heptanoic acid	–	ESI−	312.3418	2.557	2.337	*P* > 0.05
9	2,6-Dimethylheptanoyl carnitine	–	ESI+	302.2532	0.284	0.240	*P* > 0.05
10	Acetyl-l-carnitine	–	ESI−	204.1278	2.666	5.762	*P* < 0.01
11	Lysophosphatidylcholine (lysoPC)	9008-30-4	ESI−	496.3463	7.485	5.063	*P* < 0.01
12	Glycochenodeoxycholic acid	16564-43-5	ESI+	472.3022	3.718	4.745	*P* < 0.01
13	2-Chlorophenylalanine (Internal standard)	–	ESI−	199.6231			

Compared with healthy group significant difference, *P* is 0.01 < *P* < 0.05 for the significant difference, *P* < 0.01 for the extremely significant difference, *P* > 0.05 for no difference.

### Experimental methods

#### Sample collection

In the early morning and under fasting conditions, three milliliters of peripheral venous blood were collected from each participant into a standard disposable glass tube. Within one hour, the blood samples were centrifuged at 3500 rpm for 5 min to collect and seal 1.5 mL serum in an Eppendorf tube, with the specimen information marked on the tube surface. The serum samples were stored at − 80 °C for later use.

#### Pre-test process

A total of 255 serum samples of different groups were thawed at room temperature, followed by collecting 100 µL of serum and adding 20 µL of the internal standard solution (0.1 mg/mL of 2-chlorophenylalanine) and 300 µL of a 3:1 methanol/acetonitrile solution (precooled at 4 °C for half an hour before usage). The serum mixtures were vortexed for 2 min, followed by 1 min sonication. After cooling at − 20 °C, the serum mixture was thawed at room temperature and then centrifuged at 5000 rpm for 5 min at 4 °C. The supernatant was collected and filtered through a 0.22-μm filter for later experiments.

#### Required instrument and conditions for detection

##### Chromatographic conditions

An Endeavorsil MT C18 column (50 × 2.1 mm, 1.8 µm) was used, with the column temperature defaulted as room temperature. The mobile phase of the column was 0.1% formic acid aqueous solution (A)-acetonitrile (B), with a 0.30 mL/min flow rate and 5 µL injection volumes. Table S1 shows the gradient elution program.

##### Mass spectrometry conditions

Ion source: ESI positive and negative ions were scanned simultaneously, with the scanning range of MS1 m/z 100–1000 and MS2 m/z 50–550, the CDL temperature of 200 °C, and the heating module temperature of 200 °C. The drying gas flow rate was 10 L/min, and the atomizing gas flow rate was 1.5 L/min. The ion source voltages were + 4.5 kV positive ion mode and − 3.5 kV negative ion mode. The detector voltage was 1.70 kV, and the CID parameter was 50% collision energy. The mass calibration method based on automatic tuning was used to optimize voltage and the standard external method to calibrate the mass.

### Data processing and statistical analysis

Serum samples from six groups, including the HBV carrier group, chronic hepatitis B group, CHC group, DHC group, primary HCC group, and the healthy control group were tested to obtain the data of the metabolite composition and contents. IBM SPSS 24.0 software (IBM SPSS Inc., Chicago, IL) were used for statistical analysis in this study. A non-parametric test was used to analyze the difference between groups. The diagnostic values of metabolites were analyzed by the ROC curve.

### Statement

A total of people’s blood was obtained from the 980th Hospital of Chinese People’s Liberation Army Joint Logistic Support Force (Hebei Province, China) in this study and the involving human participants were approved by the Ethics Committee of Hebei Medical University. All research was performed in accordance with relevant guidelines/regulations, informed consent was obtained from all participants and/or their legal guardians.

## Results

### Differences in the four metabolites in the serum of patients with liver diseases at different stages

**Figure 1:**
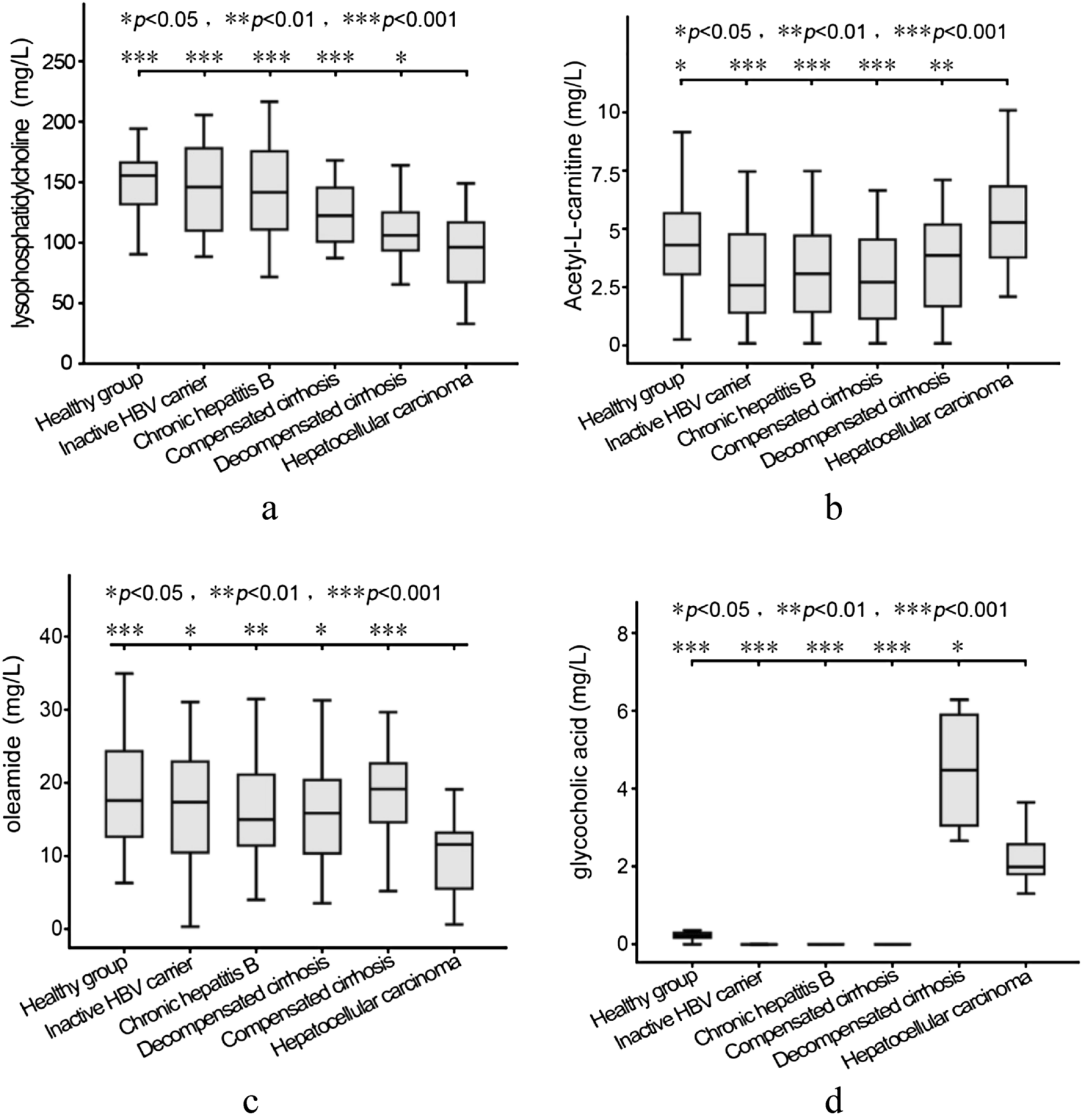
Differences in the four metabolites in the serum of patients with liver diseases at different stages. (**a**) Box plot of lysophosphatidylcholine, (**b**) Box plot of Acetyl-l-carnitine, (**c**) Box plot of Oleamide, (**d**) Box plot of glycocholic acid.

The serum lysoPC, oleic acid amide, and glycocholic acid of the HCC group were lower than those in the healthy control, HBV-carrier, hepatitis, CHC, and DHC groups. The glycocholic acid contents of the healthy control, HBV-carrier, hepatitis, and CHC were extremely low, i.e., close to 0 mg/L. The serum acetyl-l-carnitine content of the HCC group was higher than in the other groups. Table 4 and Fig. 1 show the statistical description. LysoPC is secreted by the liver and has many important physiological functions. Patients with more severe liver diseases had lower blood lysoPC content. When the liver cells were injuried, the metabolism of oleic acid amide was affected, leading to reduced content. The increased concentration of glycocholic acid suggested hepatocyte dysfunction and obstruction of hepatobiliary circulation. The high content of acetyl-l-carnitine in the HCC group was related to the high metabolic state of HCC.

The changes in serum lysoPC in the patients with liver diseases at different stages were compared with the HCC group, the means and medians of different groups were gradually decreased with the aggravation of the liver diseases. The serum lysoPC of the HCC group was significantly lower than that of the DHC group (*P* < 0.05) and the healthy control, HBV-carrier, hepatitis, and CHC groups (*P* < 0.001) (Table 3; Fig. 1a). The changes in serum acetyl-l-carnitine in the patients with liver diseases at different stages were compared with the HCC group. The serum acetyl-l-carnitine of the HCC group was higher than that of the DHC group (*P* < 0.01) the HBV-carrier, hepatitis, and CHC groups (*P* < 0.001), and the healthy control group (*P* < 0.05) (Table 3; Fig. 1b). The changes in serum oleic acid amide in the patients with liver diseases at different stages were compared with those of the HCC group, showing that the serum oleic acid amide content of the HCC group was significantly lower than that of the DHC and healthy control groups (*P* < 0.001), the hepatitis group (*P* < 0.01), and the CHC and HBV-carrier groups (*P* < 0.05) (Table 3; Fig. 1c). The changes in serum glycocholic acid in patients with liver diseases at different stages were compared with the HCC group. The serum glycocholic acid content of the HCC group was significantly lower than that of the DHC group (*P* < 0.05). The serum glycocholic acid contents of the healthy control, HBV-carrier, hepatitis, and CHC groups were extremely low and close to 0 mg/L (Table 3; Fig. 1d).

**Table 3:** Statistical descriptives of lysophosphatidylcholine, acetyl-l-carnitin, oleamide and glycocholic acid: mean ± std. deviation.

Grouping	Mean ± standard deviation (mg/L)			
	LysoPC	Acetyl-l-carnitine	Oleic acid amide	Glycocholic acid
Healthy group (n = 58)	149.81 ± 24.07	4.32 ± 1.97	28.34 ± 7.31	0.19 ± 0.12
HBV-IaC (n = 28)	146.06 ± 35.04	3.05 ± 1.96	26.65 ± 8.94	0
CHB (n = 46)	141.78 ± 38.68	3.13 ± 2.01	26.35 ± 6.66	0
CHC (n = 28)	125.07 ± 25.76	2.86 ± 1.98	26.19 ± 7.15	0
DHC (n = 47)	110.74 ± 25.67	3.68 ± 1.96	29.01 ± 5.89	4.47 ± 1.71
HCC (n = 34)	92.62 ± 32.29	5.39 ± 2.16	20.40 ± 5.19	2.22 ± 0.76

### Results of ROC curve analysis of metabolites for HCC diagnosis

This study covered liver diseases at different stages after HBV infection. To investigate the diagnostic efficacy of the above three metabolites on HCC, comparisons of the ROC curves between different liver diseases and HCC were analyzed. While reading Table 4, which shows the summary of the comparison results, it should be kept in mind that the larger the area under the curve (AUC), the higher the diagnostic accuracy.

**Table 4:** The AUC value, sensitivity and specificity of the best cutoff and the cutoff value of the largest AUC, three metabolites.

Group	LysoPC	Acetyl-l-carnitine	Oleic acid amide
	Area under the curve value, (sensitivity, specificity)(%), best cut-off value (mg/L)		
HG–HCC	0.934 (88.4, 83.3)129.1	0.646 (61.1, 82.4)	0.845 (88.6, 70.6)
IaC–HCC	0.809 (88.9, 66.7)132.9	0.790 (61.1, 88.9)	0.649 (88.9, 55.6)
CHB–HCC	0.792 (88.9, 61.1)	0.795 (100, 47.2)2.1	0.752 (88.9, 58.3)
HC–HCC	0.673 (50.0, 82.4)	0.782 (61.1, 82.4)	0.815 (88.9, 70.6) 23.6

**Figure 2:**
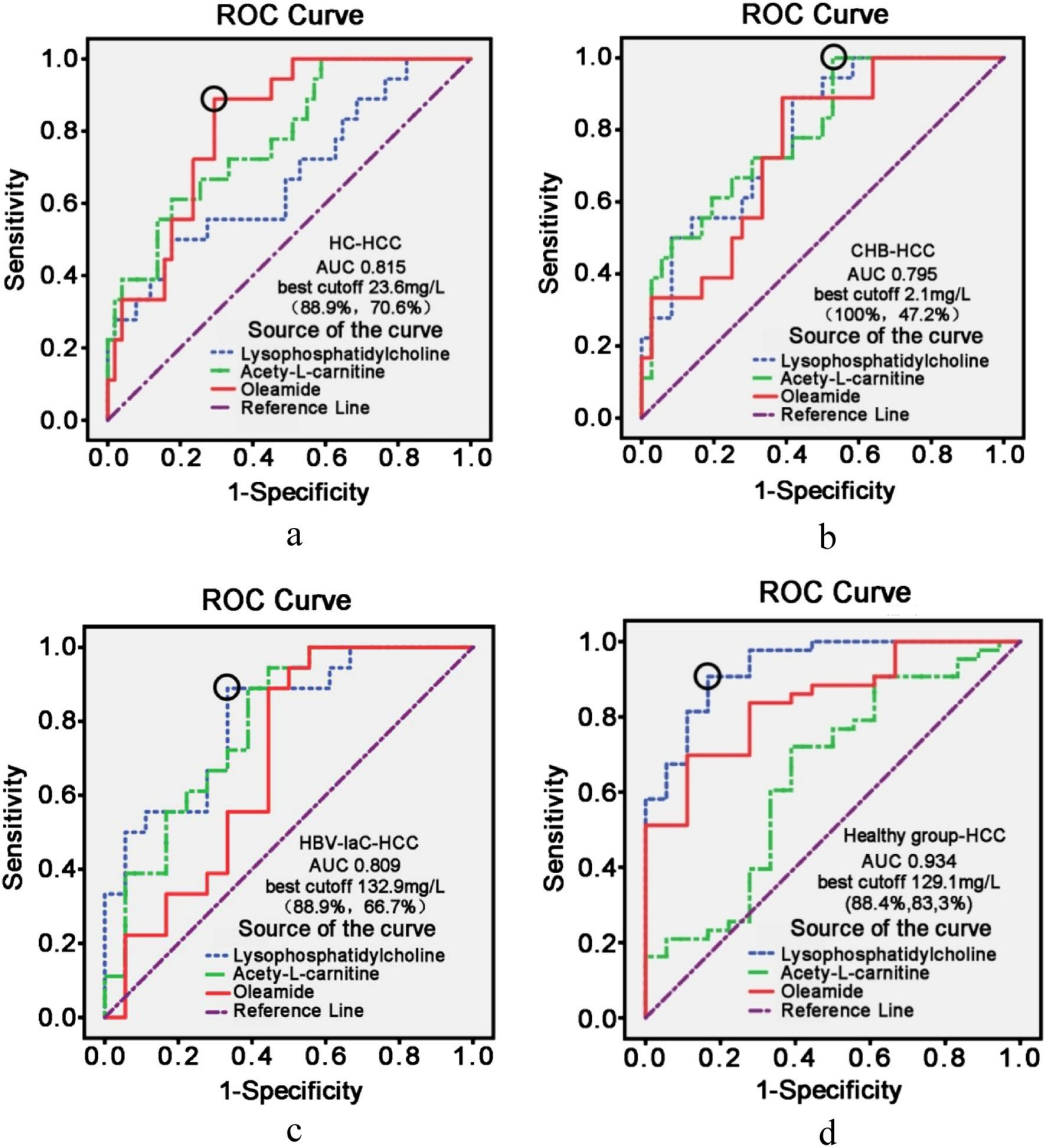
Results of the ROC curve analyses of the diagnostic values of different metabolites for HCC. (**a**) Hepatic cirrhosis-hepatocellular carcinoma. The ROC curve provided the largest AUC of 0.815 and the sensitivities and specificities reached 88.9% and 70.6%, respectively. (**b**) Chronic hepetitis B-hepatocellular carcinoma. The results of ROC curve: 0.792, 0.795, and 0.752. (**c**) Inactive HBV carrier-hepatocellular carcinoma. The ROC curve provided the largest AUC of 0.809 and the sensitivities and specificities reached 88.9% and 66.7%, respectively. (**d**) Healthy group-hepatocellular carcinoma. The ROC curve provided the largest AUC of 0.934 and the sensitivities, and specificities reached 88.4% and 83.3%, respectively.

As shown in Fig. 2a, in the analysis results between the cirrhosis groups and HCC group, the AUCs of lysoPC, acetyl-l-carnitine, and oleic acid amide ROC curves were 0.673, 0.782, and 0.815, respectively. The optimal cut-off value of the oleic acid amide ROC curve with the highest AUC value was 23.6 mg/L, showing the diagnostic sensitivity and specificity of this cut-off point as 88.9% and 70.6%, respectively. As shown in Fig. 2b, in the analysis results between the hepatitis group and HCC group, the AUCs of lysoPC, acetyl-l-carnitine, and oleic acid amide ROC curves were 0.792, 0.795, and 0.752, respectively, showing very similar diagnostic efficacies among the three substances. The optimal cut-off value of acetyl-l-carnitine was 2.04 mg/L, showing a diagnostic sensitivity and specificity of this cut-off point as 100% and 47.2%, respectively. In Fig. 2c the analysis results between the HBV-carrier and HCC groups are reported, the AUCs of lysoPC, acetyl-l-carnitine, and oleic acid amide ROC curves being 0.809, 0.790, and 0.691, respectively. The optimal cut-off value of lysoPC was 132.85 mg/L, showing that the diagnostic sensitivity and specificity of this cut-off point were 88.9% and 66.7%, respectively. As shown in Fig. 2d about the analysis results between the healthy control and HCC groups, the AUCs of lysoPC, acetyl-l-carnitine, and oleic acid amide ROC curves were 0.934, 0.647, and 0.845, respectively. The optimal cut-off value of oleic acid amide, the metabolite with the largest AUC, was 129.03 mg/L, showing a diagnostic sensitivity and specificity of 88.4% and 83.35, respectively. The lysoPO had the relatively largest AUC, with greater discriminating ability and diagnostic value between two groups, suggesting that lysoPO may be a diagnostic marker for primary HCC.

## Discussions

LysoPC is mainly secreted into the bloodstream by the liver. It is an important member of the phospholipid cycle in the liver, and it participates in various physiological functions in the body as an important product of the phospholipid metabolism. It has been reported in the literature^23^ that lysoPC belongs to a class of cytokines involved in the inflammatory responses. Millimolar concentrations of lysoPC are sufficient to stimulate the chemotaxis of T cells, neutrophils, and eosinophils^24^, stagnating the blood flow of the microcirculation and leading to tissue necrosis and organ injury^25^. After HBV infection, this injury also occurs in the liver. As the degree of liver injury increases, the content of lysoPC is reduced. Interleukin (IL)-6 upregulates the gene expression of lysoPC acyltransferase 1-4 (lysoPCAT1-4), suggesting that the concentration of cytokines is positively correlated with the degree of liver injury. Notably, the increase of IL-6 may lead to increased production of lysoPCAT1-4, which increases the conversion to lecithin, resulting in a decrease of the lysoPC content in the blood.

LysoPC can be used not only to distinguish between HCC and chronic liver diseases^25^, but also to help determine the stages of HCC progression and the metabolism of HCC patients. In this study, the means and medians of lysoPC of different groups were gradually decreased as the conditions worsened. The lysoPC contents of the patients with HBV infection in different groups were lower than the lysoPC content of the healthy control group, with the HCC group showing the lowest value. Studies have shown that the serum IL-6 content of the HCC patients is significantly elevated. Therefore, the serum lysoPC contents of the HCC patients in this study were significantly reduced. The significant reduction of lysoPC content and disruption of phospholipid metabolism may also be associated with activated inflammatory signaling pathways in the liver. As a potential marker, lysoPC also provided some reference information for the study of phospholipid metabolites at different stages of liver disease progression after HBV infection.

In 1905, Russian scientists discovered carnitine in beef. Carnitine is mainly absorbed in the intestine, and it enters the liver through the portal vein. After the activation of carnitine acetyltransferase, carnitine is catalyzed to form an acetyl group to produce acetyl-l-carnitine. In the development of cranial nerves, acetyl-l-carnitine exerts a pseudo-acetylcholine-like effect. Reduction of the carnitine level in the body may result in clinical manifestations such as progressive cardiomyopathy, skeletal myopathy, hypoglycemia, and hyperammonemia. Carnitine supplementation in patients with liver diseases, especially l-carnitine, regulates the blood ammonia concentration of the patients. In this study, the serum acetyl-l-carnitine content was significantly reduced in the patients with HBV infection when their disease progressed from chronic hepatitis to CHC. This significant difference also occurred between the healthy and the HBV-carrier groups. In addition, the serum acetyl-l-carnitine content was significantly elevated when the disease progressed to the HCC stage, suggesting that it may be related to the high metabolic state of the cancer cells. The detailed metabolic mechanism still needs further investigation.

As early as 1989, a prototype long-chain fatty acid amide lipid messenger, oleic acid amide, also known as oleamide, was discovered in the cerebrospinal fluid of sleep-deprived cats. Multiple studies have shown that oleamide induces physiological sleep in animals. The changes in serum amides in the patients were notable in the DHC patients and HCC patients, which showed a more significant reduction compared with the other groups. The results indicated that the liver may be involved in the synthesis or catabolism of amides. The above chemical reactions are affected when liver cells are injuried^26^. The concentration of amides in the serum of patients had a tendency to increase when the condition was mild, and the indicator tended to decrease as the condition was aggravated.

Cholesterol is converted into primary bile acids, including cholic acid (CA) and chenodeoxycholic acid (CD-CA), in a series of complex enzymatic reactions in liver cells. CA binds to glycine to form a type of conjugated CA compounds known as cholyglycines (CG), also called glycocholic acid. The normal liver effectively uptakes glycocholic acid. When the liver cells are injuried, or cholestasis occurs, an imbalance in the glycocholic acid uptake and excretion in the liver leads to an increase in the amount of serum glycocholic acid. Measurement of the serum glycocholic acid content sensitively evaluates the liver cell function and substance circulation in the hepatobiliary system. The results of this study showed that serum glycocholic acid was significantly increased in the patients with DHC and HCC. The existing research suggests that the continuous elevation of serum glycocholic acid concentration in the patients with hepatitis B to levels higher than normal indicates that the patients may have substantial liver injury. The recovery of the serum glycocholic acid indicator may be used as a criterion for the recovery of liver function and a criterion for the curability of the liver diseases, indicating that the patient may be discharged. The changes in the serum glycocholic acid indicator may be used to set up the follow-up plan for disease monitoring in the patients with liver diseases.

The ROC curve is based on a series of different two-category methods with the true positive rate (sensitivity) as the Y-axis and the false positive rate (1-specificity) as the X-axis. The curve is used to evaluate the discriminant effect and experimental performance; the higher the AUC, the higher the diagnostic accuracy. The ROC curves obtained in this study showed that different metabolites had certain diagnostic values for the diagnosis of HCC. Some of them showed better performance in the diagnostic experiment than others. Through the corresponding formula, the optimal cut-off value of each metabolite was obtained, showing the sensitivity and specificity of the corresponding cut-off value. Results of the ROC curve analysis showed that lysoPC had the largest AUC, i.e., the greatest discriminative ability between two groups and diagnostic value, suggesting that lysoPC may be a diagnostic marker for primary HCC. Further investigation should focus on lysoPC by conducting a large-size study to determine the diagnostic efficacy of lysoPC in HCC and to test the sensitivity and specificity of the lysoPC assay systematically.

## Conclusions

A new method based on UFLC-IT-TOF MS technology was established by optimizing the pretreatment method of mixed serum samples from liver cancer patients and healthy groups, which meets the requirements of high-throughput methods for screening metabolomics components and is suitable for the organisms being studied Samples were screened for large-scale metabolomics studies. Hemolyzed lecithin, acetylcarnitine, and oleamide are expected to become metabolic markers of liver cancer. Among them, the content of lysolecithin in the blood of patients with liver cancer was significantly higher than that of other groups. Compared with the other two metabolites, the AUC value was the largest and the diagnostic efficacy was good. It provided a reference metabolism for clinical diagnosis and scientific research.

## Supplementary Information

Supplementary Table.
