# Supplementary material for: Clinical significance of small molecule metabolites in the blood of patients with different types of liver injury
Source: Sci Rep. 2021 Jun 2;11:11642. doi: 10.1038/s41598-021-91164-9 (PMC8172926; doi:10.1038/s41598-021-91164-9)
Supplement: Supplementary file 1 — Supplementary Table. [file 41598_2021_91164_MOESM1_ESM.docx]

**Table S1 Gradient elution program of UFLC**

| Time（min） | Mobile phase A（%） | Mobile phase B（%） |
| --- | --- | --- |
| 0.01 | 85 | 15 |
| 5 | 85 | 15 |
| 30 | 50 | 50 |
| 45 | 40 | 60 |
| 55 | 40 | 60 |
| 55.01 | 85 | 15 |
